# Association between *IL7R* polymorphisms and severe liver disease in HIV/HCV coinfected patients: a cross-sectional study

**DOI:** 10.1186/s12967-015-0577-y

**Published:** 2015-06-30

**Authors:** María Guzmán-Fulgencio, Juan Berenguer, María A Jiménez-Sousa, Daniel Pineda-Tenor, Teresa Aldámiz-Echevarria, Pilar García-Broncano, Ana Carrero, Mónica García-Álvarez, Francisco Tejerina, Cristina Diez, Sonia Vazquez-Morón, Salvador Resino

**Affiliations:** Unidad de Infección Viral e Inmunidad, Centro Nacional de Microbiología, Instituto de Salud Carlos III (Campus Majadahonda), Carretera Majadahonda-Pozuelo, Km 2.2, Majadahonda, 28220 Madrid, Spain; Unidad de Enfermedades Infecciosas/VIH, Hospital General Universitario “Gregorio Marañón”, Madrid, Spain; Instituto de Investigación Sanitaria Gregorio Marañón (IiSGM), Madrid, Spain

**Keywords:** HIV/AIDS, Hepatic fibrosis, Chronic hepatitis C, *IL7R*, SNPs

## Abstract

**Background:**

Interleukin-7 (IL-7) is a critical factor for T cell development and for maintaining and restoring homeostasis of mature T cells. Polymorphisms at α-chain of the IL-7 receptor (IL7R or CD127) gene are related to evolution of HIV-infection, but there are no data concerning the evolution of hepatitis C virus (HCV) infection. The aim of this study was to analyze the association between *IL7R* polymorphisms and severe liver disease in HCV/HIV coinfected patients.

**Methods:**

We performed a cross-sectional study in 220 naïve patients who underwent a liver biopsy. *IL7R* polymorphisms (rs6897932, rs987106 and rs3194051) were genotyped using the GoldenGate^®^ assay. The outcome variables were: (a) liver biopsy: advanced fibrosis (F ≥ 3), severe activity grade (A3); (b) non-invasive indexes: advanced fibrosis (APRI ≥1.5 and FIB-4 ≥3.25). Logistic regression analysis was used to investigate the association between *IL7R* polymorphisms and outcome variables. This test gives the differences between groups and the odds ratio (OR) for liver disease.

**Results:**

Patients with rs6897932 CC genotype had higher likelihood of having A3 than patients with rs6897932 CT/TT (adjusted odds ratio (aOR) = 4.16; p = 0.026). Patients with rs987106 TT genotype had higher odds of having F ≥ 3 (aOR = 3.09; p = 0.009) than rs987106 AA/AT carriers. Finally, patients with rs3194051 AA genotype had higher odds of having severe liver fibrosis (F ≥ 3; APRI ≥1.5, and FIB4 ≥3.25) than patients with rs3194051 AG/GG genotype [aOR = 2.73 (p = 0.010); aOR = 2.52 (p = 0.029); and aOR = 4.01 (p = 0.027); respectively]. The CTA haplotype (comprised of rs6897932, rs987106, and rs3194051) carriers had higher odds of having F ≥ 3 (aOR = 1.85; p = 0.012), APRI ≥1.5 (aOR = 1.94; p = 0.023), and FIB4 ≥3.25 (aOR = 2.47; p = 0.024). Conversely, the CAG haplotype carriers had lower odds of having F ≥ 3 (aOR = 0.48; p = 0.011), APRI ≥1.5 (aOR = 0.48; p = 0.029), and FIB4 ≥3.25 (aOR = 0.29; p = 0.010).

**Conclusions:**

The presence of *IL7R* polymorphisms seems to be related to severe liver disease in HIV/HCV coinfected patients, because patients with unfavorable *IL7R* genotypes (rs6897932 CC, rs987106 TT, and rs3194051AA) had a worse prognosis of CHC.

## Background

Interleukin-7 (IL-7) is required for T cell development and for maintaining and restoring homeostasis of mature T cells. IL-7 is a critical factor in maintaining or inducing an effective antiviral CD4+ and CD8+ T cell responses [1]. IL-7 is implicated in the proliferation and survival of early T and B cells in the thymus and bone marrow, respectively. Besides, in the periphery, IL-7 enhances survival and proliferation of naïve and memory T cells, regulating the T cell homeostasis [2]. The responsiveness of IL-7 is dependent on expression of the IL-7 receptor, which is composed of the high-affinity α-chain (IL7Rα or CD127) and the common cytokine receptor gamma chain (CD132) [3].

In human immunodeficiency virus (HIV) infection, the interaction between IL-7 and CD127 has impact on development, survival, and proliferation of CD4+ T cells [4]. During HIV infection, IL-7 regulatory pathway is activated and IL-7 levels are increased, but it is not enough to maintain T cell homeostasis due to progressive destruction of CD4+ T cell [5]. In hepatitis C virus (HCV) infection, hepatocytes stimulated by type I interferon (IFN) are able to produce IL-7 and eventually lead to viral clearance and disease resolution in the liver [6]; and the early expression of CD127 on HCV-specific T cells predicts the HCV clearance during acute HCV infection [7]. However, IL-7 and CD127 levels are significantly diminished during chronic HCV infection, leading to an impaired HCV-specific cytotoxic T cell reactivity [8, 9].

The IL7Rα (CD127) is encoded by *IL7R* gene, which plays an essential role in several human diseases. Thus, *IL7R* polymorphisms have been studied in several infectious diseases [10, 11] and autoimmune diseases [12], showing a key role in the evolution of patients. In HIV infection, *IL7R* polymorphisms have been associated with the decline in the CD4+ cell count in untreated HIV-infected subjects [13], and CD4+ T-cell recovery in HIV-infected patients on combination antiretroviral therapy (cART) [14]. In HCV infection, we have recently reported an association between *IL7R* polymorphisms and virological response to HCV therapy with pegylated interferon alpha plus ribavirin (pegIFNα/ribavirin) [15]. Thus, *IL7R* polymorphisms seem to play a crucial role in the physiopathology of these diseases.

The cART has made of HIV infection a chronic manageable disease in high income countries [16]. In this setting, chronic hepatitis C (CHC) has turned into an important comorbidity and a major cause of death in HIV/HCV coinfected patients [17–19], since HIV infection accelerates the natural history of CHC and increases liver-related morbidity and mortality [20–22]. Interestingly, uncontrolled HIV replication and low CD4 counts are both associated with accelerated liver fibrosis progression in HIV/HCV coinfected patients [23], suggesting that earlier use of antiretroviral therapy could ameliorate this harmful effect [24].

The aim of this study was to analyze the association between *IL7R* polymorphisms (rs6897932, rs987106, and rs3194051) and severe liver disease in HCV/HIV coinfected patients.

## Patients and methods

### Study design and patients

We carried out a cross-sectional study in HIV/HCV coinfected patients that underwent a liver biopsy at Hospital Gregorio Marañón (Madrid, Spain) between September 2000 and November 2008. All patients were of European ancestry.

Liver biopsies were performed on patients who were potential candidates for anti-HCV therapy and had not received previous interferon therapy (naïve for HCV-treatment). Selection criteria were: no clinical evidence of hepatic decompensation, detectable HCV RNA by polymerase chain reaction (PCR), negative hepatitis B surface antigen, availability of DNA sample, CD4+ lymphocyte count higher than 200 cells/µL, and stable cART for at least 6 months before study entry or no need for cART according to treatment guidelines used in the study period [25, 26]. Patients with active opportunistic infections, active drug addiction, and other concomitant severe diseases were excluded. Thus, from our cohort of 361 HIV/HCV coinfected patients with liver biopsy data, only 220 patients had data available of *IL7R* genotypes.

The study was conducted in accordance with the Declaration of Helsinki and patients gave their written consent for the study. The Institutional Review Board and the Research Ethic Committee of the Instituto de Salud Carlos III approved the study.

### Epidemiological and clinical data

Clinical and epidemiological data were obtained from medical records. Consumption of more than 50 g of alcohol per day for at least 12 months was considered as a high intake. Body mass index (BMI) was calculated as the weight in kilograms divided by the square of the height in meters. The duration of HCV infection for patients with a history of intravenous drug use (IDU; 87.7% of patients) was estimated starting from the first year they shared needles and other injection paraphernalia, which are the most relevant risk practices for HCV transmission [27]. The duration of HCV infection was not calculated when the date of initiation of their HCV infection could not be determined with certainty (n = 19).

Biochemistry panel was measured using an autoanalyzer Hitachi 912 (Boehringer Mannheim, Germany), while patients were fasting. The degree of insulin resistance was estimated for each patient using the homeostatic model assessment (HOMA) [28]: fasting plasma glucose (mmol/L) times fasting serum insulin (mU/L) divided by 22.5 [28]. Non-invasive liver fibrosis indexes were calculated according to the formula originally described for the aspartate aminotransferase to platelet ratio index (APRI): aspartate aminotransferase (AST) [U/L]/upper limit of normal for AST (ULN)/(platelet [10^9^/L]) × 100 [29] and the FIB-4 index: age [years] × AST [U/L]/(platelets [10^9^/L]) × (alanine aminotransferase (ALT) [U/L])^1/2^) [30]. Although these indexes may have a poor ability to identify patients with intermediate stage of fibrosis [31], we have previously reported that the diagnostic performance of APRI and FIB-4 indexes were acceptable for diagnosis of advanced fibrosis in HIV/HCV-coinfected patients [32].

### HCV assays

HCV infection was documented in all patients by enzyme-linked immunosorbent assay and PCR test. HCV genotype was determined by hybridization of biotin-labeled PCR products to oligonucleotide probes bound to nitrocellulose membrane strips (INNO-LiPA HCV II, Innogenetics, Ghent, Belgium). Plasma HCV-RNA viral load was measured by PCR (Cobas Amplicor HCV Monitor Test, Branchburg, NJ, USA) and real-time PCR (COBAS AmpliPrep/COBAS TaqMan HCV test); and results were reported in terms of international units per milliliter (IU/mL), with a lower limit of detection of 10 IU/mL.

### Liver biopsy

Liver biopsies were performed as we described previously [32]. The samples were always evaluated by the same pathologist, who was unaware of the patients’ clinical or laboratory data. Liver fibrosis and necroinflammatory activity were estimated according to Metavir score as follows [33]: F0, no fibrosis; F1, mild fibrosis; F2, significant fibrosis; F3, advanced fibrosis; and F4, definite cirrhosis. The degree of necroinflammation (activity grade) was scored as follows: A0, no activity; A1, mild activity; A2, moderate activity; A3, severe activity.

### Genotyping of *IL7R* polymorphisms

We have analyzed the most common single nucleotide polymorphisms (SNPs) including at *IL7R* gene, using the databases of HapMap Project (http://snp.cshl.org/cgi-perl/gbrowse/hapmap_B35/) and NCBI (dbSNP) (http://www.ncbi.nlm.nih.gov/entrez/). The selection criteria were: (1) SNPs located at a putative regulatory region, although their effect has not been tested or demonstrated. (2) Allelic frequency greater than 20% in European people, because it is easier to detect a modest individual effect with a high SNP frequency >20% in the population when small sample sizes are used [34]. (3) A tag-SNP was selected when more than 1 SNP per gene was found and they had high linkage disequilibrium (LD). In summary, three SNPs [rs987106 (intronic region), rs6897932 (exon 6) and rs3194051 (exon 8)] were used for the genetic association study.

Genomic DNA was extracted from peripheral blood with Qiagen kit (QIAamp DNA Blood Midi/Maxi; Qiagen, Hilden, Germany). DNA samples were genotyped at the Spanish National Genotyping Center (CeGen; http://www.cegen.org/) using GoldenGate^®^ assay with VeraCode^®^ Technology (Illumina Inc. San Diego, CA, USA) [35].

### Outcome variables

We analyzed several outcome variables related to severity of liver disease [31]: (a) liver biopsy: advanced fibrosis (F ≥ 3), severe activity grade (A3); (b) non-invasive indexes: advanced fibrosis (APRI ≥1.5 and FIB-4 ≥3.25). These CHC-related outcomes were developed after a minimum follow-up time of 10 years with HCV infection.

### Statistical analysis

For the description of the study population, p values were estimated with Chi square test for categorical variables.

The genetic analysis was carried out according to additive, recessive and dominant models, selecting the model that best fitted the outcome variable analyzed in each case. The genetic association study was performed via logistic regression analysis. This test gives the differences between groups and the odds ratio (OR) for liver disease. Each regression analysis was always adjusted by the most significant co-variables associated with each one of the outcome variables, avoiding the over-fitting of the regression. The co-variables were selected by “Stepwise” algorithm (at each step, factors were considered for removal or entry: a p value for entry and exit of 0.15 and 0.20, respectively), including gender, age, alcohol intake, BMI, HOMA, nadir CD4+ T-cells, AIDS, undetectable HIV-RNA (<50 copies/ml), CD4+ T-cells, time of HCV infection, time on cART, type of cART, HCV-RNA ≥500,000 IU/ml, and HCV genotype. The percentage of patients that were excluded in each of the multivariable analyses due to incomplete data of covariates was always <5%.

These analyses were performed by using the IBM SPSS Statistics for Windows, Version 21.0 (IBM Corp, Chicago, Armonk, NY, USA). In addition, pair-wise LD and Hardy–Weinberg equilibrium (HWE) analyses were computed by Haploview 4.2 software. Haplotype-based association testing was performed using Plink software (http://pngu.mgh.harvard.edu/~purcell/plink/) and each haplotype was compared with the rest of haplotypes (there was no reference category). All p values were two-tailed and statistical significance was defined as p < 0.05.

## Results

### Characteristics of the patients

Table 1 shows the epidemiological and clinical characteristics of 220 HIV/HCV coinfected patients.

**Table 1:** Clinical and epidemiological characteristics of HIV/HCV-coinfected patients

Characteristics	All patients
No. (%)	220 (100%)
Male, n (%)	162 (73.6%)
Age, years	39.8 (37.4; 44)
HIV acquired by IDU, n (%)	193 (87.7%)
Years since HCV infection	21.3 (17.1; 24.4)
Prior AIDS, n (%)	60 (27.3%)
cART, n (%)	183 (83.2%)
Time on cART, years	4.4 (2.5; 6.7)
Current cART protocols, n (%)	
PI-based	50 (22.7%)
NNRTI-based	114 (51.8%)
NRTI-based	129 (58.6%)
Metabolic markers	
BMI, kg/m^2^	22.4 (20.8; 24.6)
BMI ≥25 kg/m^2^	50 (22.9%)
HOMA	2.10 (1.27; 3.73)
HOMA ≥3	71 (33.5%)
HIV markers	
Nadir CD4+, T cells/μL	192 (84; 318)
CD4+, T cells/μL	467 (324; 672)
HIV-RNA <50 copies/mL, n (%)	162 (73.6%)
HCV markers, n (%)	
HCV-RNA ≥500.000 IU/ml	162 (74.7%)
HCV genotype	216 (98.2%)
HCV-GT1	123 (55.9%)
HCV-GT2	5 (2.3%)
HCV-GT3	50 (22.7%)
HCV-GT4	38 (17.3%)
Metavir score, n (%)	
Significant fibrosis (F ≥ 2)	109 (49.5%)
Advanced fibrosis (F ≥ 3)	49 (23.3%)
Cirrhosis (F4)	23 (10.5%)
Moderate activity grade (A ≥ 2)	114 (52.8%)
Severe activity grade (A3)	24 (11.1%)
Fibrosis indexes	
APRI	0.75 (0.45; 1.30)
APRI ≥1.5	36 (17.2%)
FIB-4	1.43 (1.03; 2.03)
FIB-4 ≥3.25	20 (9.6%)

Values expressed as absolute numbers (%) and median (percentile 25; percentile 75).

*AIDS* acquired immunodeficiency syndrome, *APRI* aspartate aminotransferase to platelet ratio, *BMI* body mass index, *cART* combination antiretroviral therapy, *HCV* hepatitis C virus, *HCV-RNA* HCV plasma viral load, *HIV* human immunodeficiency, *HIV-RNA* HIV plasma viral load, *HOMA* homeostasis model assessment, *IDU* intravenous drug users, *NNRTI* no nucleoside analog reverse-transcriptase inhibitors, *NRTI* nucleoside analog reverse-transcriptase inhibitors, *PI* protease inhibitors.

### Characteristics of *IL7R* polymorphisms

**Figure 1:**
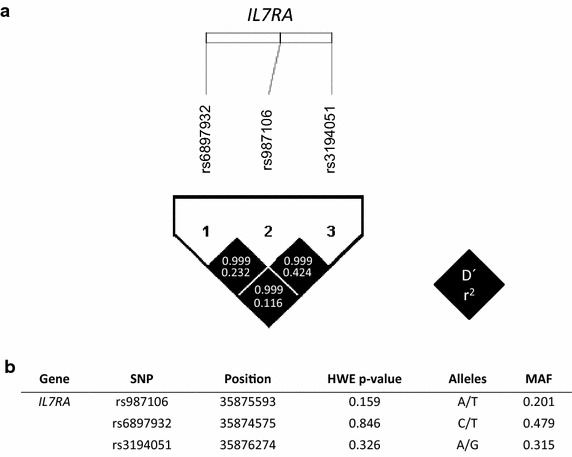
**a** Pairwise linkage disequilibrium (LD) patterns for three polymorphisms through *IL7RA* regions. Each *diagonal* represents a different SNP, with each *square* representing a pairwise comparison between two SNPs. **b** Allele and genotype frequencies, Hardy–Weinberg Equilibrium (HWE) and linkage disequilibrium (LD) for *IL7RA* polymorphisms (rs987106, rs6897932 and rs3194051) in HIV/HCV coinfected patients. *HWE* Hardy–Weinberg equilibrium, *MAF* minor allele frequency, *SNP* single nucleotide polymorphism.

A strong LD (non-random association of alleles at different loci) among *IL7R* polymorphisms was found (D′ ≥ 0.999), meaning that there is no evidence for recombination between these SNPs. However, the r-square among SNPs was low (r-square <0.50), meaning that the *IL7R* polymorphisms did not provide exactly the same information and the *IL7R* polymorphisms cannot substitute one for another (Figure 1a). Additionally, all SNPs had a minimum allele frequency (MAF) >5%, displayed missing values <5%, and were in HWE (p > 0.05) (Figure 1b).

### *IL7R* polymorphisms and severe liver disease

For rs6897932 polymorphism (Table 2A), patients with rs6897932 CC genotype had higher frequency of A3 in liver biopsy (p = 0.023) and had higher likelihood of having A3 than patients with rs6897932 CT/TT [adjusted odds ratio (aOR) = 4.16; p = 0.026].

**Table 2:** Relationship between *IL7RA* polymorphisms and severe liver disease in HIV/HCV-coinfected patients

	Unadjusted			Adjusted	
	CT/TT	CC	P^a^	aOR (95% CI)	p^b^
(A) rs6897932					
F ≥ 3	20.5% (17/83)	23.4% (32/137)	0.619	1.14 (0.54;2.32)	0.735
APRI ≥1.5	15% (12/80)	18.6% (24/129)	0.502	1.14 (0.48;2.68)	0.770
FIB4 ≥3.25	10% (8/80)	9.3% (12/129)	0.868	0.80 (0.25;2.56)	0.705
A3	4.9% (4/82)	14.9% (20/134)	*0.023*	4.16 (1.19;14.59)	*0.026*

	Unadjusted			Adjusted	
	AA/AT	TT	P^a^	aOR (95% CI)	p^b^
(B) rs987106					
F ≥ 3	18.8% (32/170)	34.7% (17/49)	*0.019*	3.09 (1.32;7.22)	*0.009*
APRI ≥1.5	14.3% (23/161)	27.7% (13/47)	*0.033*	1.84 (0.72;4.70)	0.201
FIB4 ≥3.25	7.5% (12/161)	17% (8/47)	*0.050*	2.33 (0.67;8.05)	0.181
A3	10.1% (17/168)	14.9% (7/47)	0.358	1.82 (0.63;5.23)	0.269

	Unadjusted			Adjusted	
	AG/GG	AA	P^a^	aOR (95% CI)	p^b^
(C) rs3194051					
F ≥ 3	16.7% (20/120)	29.3% (29/99)	*0.026*	2.73 (1.22;5.87)	*0.010*
APRI ≥1.5	11.5% (13/113)	24.2% (23/95)	*0.016*	2.52 (1.10;5.77)	*0.029*
FIB4 ≥3.25	5.3% (6/113)	14.7% (14/95)	*0.022*	4.01 (1.17;13.71)	*0.027*
A3	12.6% (15/119)	9.4% (9/96)	0.455	0.79 (0.30;2.10)	0.634

Statistically significant differences are shown in italics.

*HCV* hepatitis C virus, *HIV* human immunodeficiency virus, *CHC* chronic hepatitis C, *95% CI* 95% of confidence interval, *aOR* adjusted odds ratio, *p value* level of significance, *A3* severe activity grade (Metavir), *F* *≥* *3* advanced liver fibrosis (Metavir), *APRI* aspartate aminotransferase to platelet ratio index.

^a^p values were calculated by Chi squared test.

^b^p values were calculated by multivariate logistic regression adjusted by the most important clinical and epidemiological characteristics (see “Statistical analysis” section).

For rs987106 polymorphism (Table 2B), patients with rs987106 TT genotype had higher frequency of severe fibrosis (F ≥ 3, APRI ≥1.5, and FIB4 ≥3.25) than patients with rs987106 AA/AT genotype (p = 0.019, p = 0.033, and p = 0.050; respectively). Furthermore, rs987106 TT genotype was related to higher odds of having F ≥ 3 (aOR = 3.09; p = 0.009).

For rs3194051 polymorphism (Table 2C), patients with rs3194051 AA genotype had higher frequency of F ≥ 3 (p = 0.026), APRI ≥1.5 (p = 0.016), and FIB4 ≥3.25 (p = 0.022). Besides, patients with rs3194051 AA genotype had higher odds of having severe liver fibrosis (F ≥ 3; APRI ≥1.5, and FIB4 ≥3.25) than patients with rs3194051 AG/GG genotype [aOR = 2.73 (p = 0.010); aOR = 2.52 (p = 0.029); and aOR = 4.01 (p = 0.027); respectively].

Three major haplotypes (comprised of rs6897932, rs987106, and rs3194051) were found (Table 3): CTA, CAG, and TAA. The CTA haplotype carriers had higher odds of having F ≥ 3 (aOR = 2.01; p = 0.007), APRI ≥1.5 (aOR = 1.98; p = 0.028), and FIB4 ≥3.25 (aOR = 2.69; p = 0.023). Conversely, CAG haplotype carriers had lower odds of having F ≥ 3 (aOR = 0.42; p = 0.004), APRI ≥1.5 (aOR = 0.45; p = 0.026), and FIB4 ≥3.25 (aOR = 0.24; p = 0.007).

**Table 3:** Association of *IL7RA* haplotypes (rs987106, rs6897932 and rs13126816) with severe liver disease in HIV/HCV coinfected patients

*IL7RA* haplotypes					Adjusted	
rs6897932	rs987106	rs3194051	Freq.	Outcomes	aOR (95% CI)	p value
C	T	A	47.9%	F ≥ 3	2.01 (1.19;3.40)	*0.007*
				APRI ≥1.5	1.98 (1.06;3.71)	*0.028*
				FIB4 ≥3.25	2.69 (1.10;6.60)	*0.023*
				A3	NA	–
C	A	G	31.5%	F ≥ 3	0.42 (0.22;0.78)	*0.004*
				APRI ≥1.5	0.45 (0.22;0.94)	*0.026*
				FIB4 ≥3.25	0.24 (0.07;0.76)	*0.007*
				A3	NA	–
T	A	A	20.1%	F ≥ 3	0.86 (0.44;1.67)	0.647
				APRI ≥1.5	0.92 (0.42;2.04)	0.838
				FIB4 ≥3.25	1.07 (0.35;3.26)	0.904
				A3	NA	–

P values were calculated by multivariate logistic regression adjusted by the most important clinical and epidemiological characteristics (see “Statistical analysis” section). Statistically significant differences are shown in italics.

*95% CI* 95% of confidence interval, *aOR* adjusted odds ratio, *p value* level of significance, *A3* severe activity grade (Metavir), *F* *≥* *3* advanced liver fibrosis (Metavir), *APRI* aspartate aminotransferase to platelet ratio index, *NA* not available due to a low number of patients in one of the groups.

## Discussion

To our knowledge, this study is the first description of the relation between *IL7R* polymorphisms and severity of liver disease in HIV/HCV-coinfected patients. The major findings of our study were: (1) *IL7R* rs6897932 CC was associated with higher odds of having severe activity necroinflammatory grade; (2) *IL7R* rs987106 TT and rs3194051 AA genotypes were related to increased odds of having severe fibrosis; and (3) *IL7R* CTA haplotype was associated with severe fibrosis while *IL7R* CAG haplotype had a protective nature. Moreover, these relationships were less evident when patients were stratified by HCV genotype (*data not shown*), perhaps due to a smaller sample size of the groups.

The rs6897932 C/T polymorphism is a missense variant located in the alternatively spliced exon 6 of *IL7R* gene, causing a substitution of threonine with isoleucine in the transmembrane domain of the *IL7R* gene. This polymorphism is implicated in splicing regulation, where the C allele leads to increased skipping of exon 6 by putatively disrupting an exonic splicing motif [36], increasing the production of sCD127 (soluble isoform) and decreasing CD127 levels (membrane-bound isoform). In this setting, rs6897932 CC genotype has been associated to higher plasma levels of sCD127 in Caucasian HIV-infected patients [14, 37], which binds to circulating IL-7 and decreases the IL-7 bioavailability, limiting its effects [38]. Thus, rs6897932 C allele has been related to slower CD4+ T-cell recovery in European HIV infected patients on cART [39]. In our study, patients with rs6897932 CC genotype had higher odds of severe necroinflammatory activity grade. Given that the *IL7R* rs6897932 CC genotype is related to increased risk of autoimmune diseases [36, 40], it is possible to speculate that higher level of sCD127 may confer worse immunological control of HCV infection in liver, with a higher necroinflammatory activity in liver biopsy. However, further studies investigating the underlying mechanisms are warranted.

In our study we also found that rs987106 TT and rs3194051 AA genotypes were related to higher odds of having severe liver fibrosis. The rs987106 polymorphism is an intronic variant between exons 6 and 7 of *IL7R* gene and rs3194051 is a missense polymorphism located at exon 8. These polymorphisms are predicted to be involved in post-transcriptional regulation and besides, rs3194051 is also predicted to be implicated in proximal transcriptional regulation of CD127, according to an in silico analysis by using rSNPBase software [41]. In regards to rs987106, previous articles have described an association between rs987106 TT genotype and higher sCD127 levels [14] and rapid AIDS progression [13]. The rs3194051 polymorphism, a tag SNP of *IL7R* haplotype 4 in European populations, has been associated with highest CD127 on CD4+ T-cell, higher frequencies of recent thymic emigrants, and lower plasma sCD127 levels in patients with multiple sclerosis [42]. Thus, the rs3194051 A allele ancestral would be associated with lower CD127 on CD4+ T-cell, lower frequencies of recent thymic emigrants, and higher plasma sCD127 levels. Furthermore, rs3194051 A allele has been associated with slower CD4+ recovery in HIV infected patients on cART [14] and lower odds of achieving sustained virological response (SVR) after pegIFNα/ribavirin in HIV/HCV coinfected patients [15].

Moreover, as noted in the preceding paragraphs, unfavorable alleles of these three *IL7R* polymorphisms (rs6897932 C, rs987106 T, and rs3194051 A) have been related to low CD4+ T-cells and rapid AIDS progression in Caucasian naïve HIV infected patients [13] and slower CD4+ recovery in patients on cART [14, 39]. Besides, uncontrolled HIV replication and low CD4 counts are both associated with accelerated liver fibrosis progression in HIV/HCV coinfected patients [23]. Thus, unfavorable *IL7R* genotypes (rs6897932 CC, rs987106 TT, and rs3194051 AA) could lead to an increased risk of severe liver disease, by decreasing CD4+ cells count and enhancing AIDS progression in HIV/HCV coinfected patients [43].

In our study, *IL7R* haplotypes (comprised of rs6897932, rs987106, and rs3194051) were also investigated to verify whether they could improve the association of severe fibrosis compared to *IL7R* polymorphisms alone. We found that CTA haplotype was associated with higher odds of having severe liver fibrosis; while CAG haplotype (comprised of one allele associated with higher sCD127 levels and two alleles related to lower sCD127 levels) was associated with lower odds of having severe liver fibrosis. However, OR values were similar to those obtained for individual SNPs. In this regard, we also recently reported that *IL7R* CTA haplotype (three alleles associated with higher sCD127 levels) was related to lower odds of achieving a SVR [15].

According to what we have discussed until now, *IL7R* polymorphisms are related to increased odds of having severity of liver disease, possibly due to regulation of sCD127 levels. Previous reports have suggested that *IL*-*7R* polymorphisms may have an influence on T-cell development and homeostasis, and thereby might contribute to an altered immune regulation [42, 44]. Moreover, the CHC progression, which includes cirrhosis, end-stage liver disease, and hepatocellular carcinoma, is more rapid in HIV-infected patients, possibly because a damaged immune system may increase the risk for CHC progression among persons infected with HIV [45–47]. Therefore, one should not rule out the possible influence of *IL7R* polymorphisms in the development of end-stage of liver disease and hepatocellular carcinoma in HIV/HCV coinfected patients.

In our study, we compared the *IL7R* genotype frequencies depending on the presence of each of the outcome variables (independent events), which generated a high number of comparisons. There is a considerable controversy about adjusting the “p value” after multiple tests on clinical-orientated studies [48, 49]. In our study there was a hypothesis supported by theory and previous reports in several infectious diseases [10, 11], including HIV infected subjects [13, 14] and HIV/HCV coinfected patients [15], and showing a key role of *IL7R* polymorphisms in the evolution of patients. Therefore, we were not literally doing a random search of a meaningful result, and our results should not be affected by the fact of carrying out a high number of statistical tests. Moreover, since p value is depending on the sample size, only big effects would be detected in small populations and it may be possible that we did not find any significant adjusted p value in some comparisons due to our size-limited population. We should take into account the fact that the effect size of our study is low since complex human diseases are under the control of many genes, each one of them contributing with modest individual effects [50].

There are other aspects to be taken into account for the correct interpretation of the results. Firstly, this is a cross-sectional study with data collected retrospectively. Secondly, the study contains a limited number of patients, which could limit achieving statistically significant p values in some groups and it may lead to potentially large percentage fluctuations in the proportion. Thirdly, all selected patients met a set of criteria for starting HCV treatment and this may have introduced a selection bias. Fourthly, this study was performed on patients with European ancestry, and it would be interesting to perform these analyses on different ethnic groups. Fifthly, our study only included HIV/HCV-coinfected patients and it would be interesting to know the role of studied *IL7R* polymorphisms in HCV monoinfected patients, but we did not have access to a cohort of HCV monoinfected patients.

## Conclusions

The presence of *IL7R* polymorphisms seems to be related to severe liver disease in HIV/HCV coinfected patients, because patients with unfavorable *IL7R* genotypes (rs6897932 CC, rs987106 TT, and rs3194051AA) had a worse prognosis of CHC.

## Abbreviations

HIV: human immunodeficiency virus
HCV: hepatitis C virus
cART: combination antiretroviral therapy
IL-7: interleukin-7
IL7R: interleukin-7 receptor-alpha gene
IL7Rα or CD127: high-affinity α-chain of IL-7 receptor
CD132: common cytokine receptor gamma chain
pegIFNα/ribavirin: pegylated interferon alpha plus ribavirin
PCR: polymerase chain reaction
BMI: body mass index
IDU: intravenous drug use
HOMA: homeostatic model assessment
APRI: aspartate aminotransferase to platelet ratio index
AST: aspartate aminotransferase
ALT: alanine aminotransferase
F ≥ 3: advanced fibrosis
A3: severe activity grade
LD: linkage disequilibrium
HWE: Hardy–Weinberg equilibrium
aOR: adjusted odds ratio
SVR: sustained virological response
